# Asthma in Infants and Young Children

**Published:** 1908-01-25

**Authors:** Edmund Cautley

**Affiliations:** Physician Belgrave Hospital for Children; Physician, Metropolitan Hospital.


### ASTHMA IN INFANTS AND YOUNG CHILDREN.

By EDMUND CAUTLEY, M.D. (Cantab.), F.R.C.P. Lond., Physician Belgrave Hospital for Children; Physician, Metropolitan Hospital.

(Abstract of a Lecture delivered at the Polyclinic Institute.)

Asthma is most often seen in the upper classes and among the Jews. It is less frequent in hot and dry climates than in low and damp ones. Asthmatic subjects may suffer from urticaria or eczema, which may be simultaneous or alternative, or may have no connection.

Cases may be of central or reflex origin. The central causes are vague and indefinite, but seem to depend on hyper-sensitiveness of the nerve centres. The reflex cases depend chiefly on a catarrhal condition of the mucous tract. This mucous membrane, whether it be in the nose or trachea or lower down the respiratory tract, is hyperæsthetic and liable to catarrh; a condition in which very slight causes will induce an attack of asthma. This explains why east winds, dust, hay-pollen, ipecacuanha, various odours of animals, etc., bring on attacks of paroxysmal sneezing or hay asthma. Gastric troubles and constipation are liable to set on an attack. Henoch has described a special gastric type under the name of "Asthma dyspepticum."

Among other reflex causes may be mentioned nasal obstruction, polypi, adenoids and enlarged tonsils, and foreign bodies in the various orifices of the body. I have not come across any case that I could definitely ascribe to the local irritation of a foreign body. Enlarged bronchial glands are another reflex cause. They may, like the enlarged thymus, give rise to paroxysmal dyspnœa, but not to true asthma. Undue importance has been attached to enlarged glands as a cause. You will hardly ever see a case of tuberculous meningitis without finding the glands enlarged, yet the patients have no attacks of paroxysmal dyspnœa.

### Pathology.

It is not a pure neurosis of the nerve centres; of the nature of an epilepsy of the respiratory centre. The chief theories held are that the paroxysm is due (1) to a spasm of the muscles of the smaller bronchi; (2) to a swelling of the mucous membrane of the smaller bronchi; or (3) to a combination of these two factors. In favour of the first view one finds that drugs that relax spasm, *e.g.*, inhalation of chloroform or amyl nitrite, injection of morphia subcutaneously, or the administration of nitrites by the mouth (supposed to act on unstriped muscle) will relieve asthma. These drugs also act on the heart as cardiac depressants. The swelling of the mucous membrane of the smaller bronchi has been ascribed to Angio-neurotic œdema, due to irritation of the terminals of the vagus and analogous to urticaria of the skin; to hyperæmia; or to vasodilatation. There is considerable evidence in favour of the view that such swelling does occur. The paroxysm is preceded by respiratory discomfort; exposure of the chest may bring it on; the inhalation of hot air may make the paroxysm worse; and it is often accompanied with general vaso-constriction elsewhere, as is shown by the pale face, lips and extremities. Many remedies that relieve a paroxysm of asthma are general vaso-dilators or local vaso-constrictors, or act as cardiac depressants.

On the whole, I think that one must take the view that the majority of these cases are due to swelling of the mucus membrane, vaso-dilator in character, which sets up a certain amount of spasmodic contraction of the bronchial tubes, and that there are also cases of pure spasm which may occur independently of such swelling. In some cases of asthma you will find swelling of the mucous membrane of the nose, and with the laryngoscope you may see that of the trachea also swollen.

### Symptoms.

As regards symptoms the cases must be divided into two classes: (*a*) those that are catarrhal in origin; (*b*) those that are spasmodic purely. The catarrhal cases are seen commonly in infancy; the pure spasmodic forms later in life and very rarely before the age of four years. The *catarrhal* cases occur in infants liable to catarrh; in the rachitic, the fat babies, and those with adenoids or enlarged tonsils predisposing to catarrhal conditions of the respiratory mucosa. In such cases the onset of the attack is preceded by definite signs of catarrh, slight in some instances. Indeed, the baby may be put to bed apparently perfectly well, more commonly breathing a little more rapidly than usual and wheezing a little. In the course of a few hours the child wakes up with great respiratory disturbance, anxious, restless, and breathing rapidly. The respiration rate varies considerably, sometimes being about 40 per minute, at other times as much as 90 per minute. The pulse is small, frequent, sometimes irregular. The chest indicates the typical condition of severe dyspnœa, similar to that of acute catarrhal bronchitis, with no special hyper-resonance, but sonorous râles, rhonchus, and sibilus, a few finer crepitations at the bases of both lungs behind, and marked recession of the lower ribs and the soft parts above the clavicles. Usually the dyspnœa persists from two to six hours or more. Generally, next morning, the dyspnœa has subsided and the child is well except for a certain amount of catarrh of the respiratory passages, and in two or three days is absolutely well. If the attack is very severe it may last very much longer, or it may occur on successive nights at just about the same time; sometimes it is diurnal, periodic in character, coming on during sleep.

Another type of case is that of a child two or three years of age, who has had an attack of broncho-pneumonia or bronchitis. After a time it develops severe dyspnœa without any special rise of temperature or increase of the chest signs. As a general rule little attention is paid to the condition, and it is thought to be part of the illness for which the child is being treated; that the dyspnœa is due to paroxysmal asthma is not realised.

In a third type sonorous râles are present, but no special dyspnœa. These attacks come on in these bronchial children periodically, lasting for a few days at a time without any special symptoms. Thus catarrhal asthma is of three types: (1) Acute, ushered in with slight catarrh; (2) occurring during the course of catarrhal pneumonia or bronchitis; (3) no actual paroxysm, but recurrent attacks of sonorous sibilant râles in the chest. Two diseases specially predispose to the catarrhal conditions, antecedent to asthma, namely, measles and whooping cough.

The *spasmodic* type of asthma occurs in older children, and is closely allied both in character and duration to that of the adult type, though it is usually less severe and more prolonged. The onset may be sudden, and this is a point in favour of the view that the attacks are not purely spasmodic in origin to start with; it is uncommon to get an attack absolutely sudden in its onset. It is preceded, for a few hours perhaps, by a feeling of tightness or constriction across the chest, a sense of suffocation, air hunger, and a little inspiratory wheezing. Then the attack comes with great severity, with dyspnœa differing from the catarrhal type. It is inspiratory in character, inspiration is violent and shallow. At the end of each inspiration there is a pause with the object of keeping the tubes open as widely as possible, followed by a very prolonged expiration. The child gets pallid as the attack reaches its maximum, and gradually cyanosed, sits up in bed with clenched hands, head thrown back, mouth open, eyes congested and staring. These symptoms are maintained at this level for a varying period.

In children it is rare to meet with attacks of this severity, rarely going so far as cyanosis, but usually lasting a longer period. Digestive troubles are particularly apt to bring on attacks of this kind in children, and they are exaggerated by damp atmosphere and by east winds.

### Diagnosis.

One has to differentiate between the paroxysmal dyspnœa of asthma and that due to other causes—a difficult task with the catarrhal type occurring in infancy, for it is so apt to simulate acute capillary bronchitis. In acute bronchitis the dyspnœa varies in severity, whereas in asthma it maintains the same degree of intensity for some time. Then, too, you will find that a very important differential point is that the paroxysmal dyspnœa of catarrhal asthma is out of proportion to the physical signs of catarrh found in the chest. In catarrhal bronchitis sufficient physical signs are found to account for the dyspnœa present. If an asthmatic attack comes on during an attack of bronchitis the physical signs are not exaggerated in proportion to the increased dyspnœa. The essential difference between catarrhal and spasmodic asthma is that in catarrhal asthma there is a severe dyspnœa in which the frequency of the respiration may amount to as much as 90 per minute. In the spasmodic form the dyspnœa is infrequent. There is great difficulty in breathing, which is slow and laboured. It may be increased in frequency in proportion to the ordinary rate of breathing, but never as much as in the case of catarrhal asthma. In catarrhal asthma there is marked recession of the chest; in spasmodic, the recession is slight or absent. In catarrhal asthma fine râles and vesicular breathing are present; in spasmodic asthma large sonorous and sibilant râles are frequent, fine râles few, and a vesicular murmur absent.

In addition, in catarrhal asthma there is no marked alteration in resonance, whereas in spasmodic asthma the note is hyper-resonant, the chest assuming the position of complete inspiration. It is fully expanded, the inspiratory muscles strongly contracted, the expiratory muscles flaccid, and the diaphragm depressed. In catarrhal asthma the chest is sucked in on account of the resistance to the entry of air. In the diagnosis of these conditions you must attach importance to the relative amount of dyspnœa in proportion to the obstruction and physical signs, and to the family history, still more to the history of recurrent attacks. It is by no means easy to diagnose asthma in a first attack, but with history of recurrence the diagnosis is simple. In older children asthmatic dyspnœa has to be diagnosed from the hysterical form, in which the child is breathing with extreme rapidity but without any distress. Laryngeal obstruction and foreign bodies in the air passages are apt to lead to respiratory distress. In that type the difficulty is purely inspiratory in character. Dyspnœa may occur from the pressure of enlarged glands. Finally, paroxysmal dyspnœa may occur in certain cardiac conditions, usually on account of the fact that the frequency of breathing is increased by exertion. Severe dypsnœa may come on after slight exertion in gastric dilatation from flatulence. It is especially apt to occur in the myocarditis of rheumatism.

### Prognosis.

With regard to the prognosis of the disease, it is extremely rare for anyone to die from an attack of asthma. An infant may die, however, from suffocation, cyanosis and prostration. Recurrence is very frequent. The prognosis depends upon several different factors. Recurrence is common in spasmodic asthma, which is much more likely than catarrhal asthma to continue throughout life. The prognosis as regards recurrence varies with the presence of an exciting cause. If there is no definite exciting cause prognosis is bad. Thus, in dealing with the catarrhal variety the prognosis would be that of the respiratory affection rather than that of the attack of asthma. If the catarrhal condition can be cured, there is a strong probability that the patient will outgrow the attacks of asthma. In recurrent attacks the prognosis depends upon the frequency of the attacks. In frequent attacks, with short intervals between them, the lung has not time to return to its normal condition, and the condition of the lung tends to become permanent. Patients are liable to get emphysema and dilatation of the right side of the heart. In the emphysematous lungs, due to recurrent attacks of catarrhal asthma, the prognosis is much worse than if the child were free from pulmonary mischief. On the whole the prognosis of asthma in children is by no means bad, although people are liable to take a gloomy view of it. It is most favourable, if an exciting cause is found, such as adenoids or enlarged tonsils, or some conditions of the nasal passages. A considerable amount of importance has been attached to the state of the nasal mucous membrane as a cause. It has been maintained that there is a small area on the triangular cartilage of the nose, which is affected by œdema, and that it is due to the irritation of this small area that the asthmatic attack is produced, and that asthma can be cured by cauterising this local patch. That is an unjustifiable claim. Many cases have been treated and some have got well. The cure of nasal obstruction does not always cure asthma, still less hay-fever. Asthma has even been set up by the removal of a polypus from the nose.

### Treatment.

The treatment is primarily preventive; secondly, the treatment of the attack; and, thirdly, the treatment of the disease between the attacks.

Preventive treatment consists in treating carefully children of asthmatic parentage. They must be reared under the best conditions of life as regards hygiene, clothing, diet and mode of life. They must be treated so as to harden the respiratory mucous membrane and to make them as little liable to catarrhal affections as possible. Catarrhal asthma must be treated as a case of catarrh. Choose a dry and warm climate with a sandy soil. Suitable climatic localities in England are Bournemouth, Hastings, and the Ascot district. High altitudes are not suitable for emphysematous cases. Spasmodic cases improve in high altitudes. Warm clothing is essential. In order to prevent the attack at night administer antipyrin, phenacetin or bromide, at bedtime. Spasmodic attacks occur at about three in the morning. In mild cases it is generally sufficient that the patient should sleep in a room with the windows open and with plenty of bedclothes. The body should be kept warm, but the air should be kept cool. Such treatment might be absolutely unsuitable for catarrhal attacks.

The diet should be nutritious, but it should not be given in large quantities at a time. Children should be taught to masticate the food thoroughly. Avoid meat extracts and an excessive quantity of meat or carbohydrates. Some cases have been treated with a purely anti-diabetic diet, but in my experience it does no good. Give an ordinary rational diet, taking care that it is not excessive in any one constituent. When the attack comes on various remedies are used, with various objects in view. Remedies are used with the object of causing general dilatation of the blood-vessels of the skin and so reducing the congestion of the respiratory mucosa. The hot bath or vapour bath, hot fomentations or turpentine stupes to the chest, inhalation of chloroform or amylnitrite, chloral by the mouth or rectum, alcohol, or even morphia subcutaneously, are useful. On the whole I do not recommend morphia for children, or even for adults, unless it is absolutely necessary. It is worth while trying if you can produce local vaso-constriction in addition, by getting the patient to inhale cold air from ice in a jug, or the fumes of brown paper, leaves of stramonium, belladonna, or hyoscyamus, or some of the proprietary mixtures such as Himrod's powder. These are all vaso-constrictors. They are extremely irritating and absolutely unsuitable for all cases of catarrhal asthma, in which warm inhalations of creosote, turpentine or the vapor pini sylvestris are more valuable.

Drugs acting as cardiac depressants may be given to reduce the mucosal congestion. Most of them are drugs which produce nausea, faintness and collapse, and some of them, like lobelia, tartar emetic, and tobacco produce no effect until they do so. In the catarrhal type good may be done by an emetic at the onset of the attack. Use the syrup of ipecacuhana, the wine, or the powder. The nasal mucous membrane may be treated with cocaine sprays, adrenalin inhalations, or cauterisation. Between the attacks recourse must be had to general principles of treatment, especially with a view of stopping catarrhal conditions. Reliance has to be placed on iodide of potassium, syrup of the iodide of iron, and cod liver oil.

Of other remedies arsenic is about the most useful tonic. Strychnine, nux vomica, and cinchona come next. The most important principle in the treatment of infantile asthma is the treatment of the catarrhal conditions and the local sources of irritation which are liable to bring on an attack rather than to postpone the treatment until it is necessary to relieve an actual spasm.

